# Using photoplethysmography data to estimate heart rate variability and its association with organ dysfunction in pediatric oncology patients

**DOI:** 10.1038/s41746-018-0038-0

**Published:** 2018-07-25

**Authors:** Anoop Mayampurath, Samuel L Volchenboum, L. Nelson Sanchez-Pinto

**Affiliations:** 1 Department of Pediatrics, The University of Chicago, Chicago, IL USA; 2 Center for Research Informatics, The University of Chicago, Chicago, IL USA; 3 Division of Critical Care Medicine, Ann & Robert H. Lurie Children’s Hospital of Chicago, Chicago, IL USA; 4 Departments of Pediatrics & Preventive Medicine, Northwestern University Feinberg School of Medicine, Chicago, IL USA

**Keywords:** Risk factors, Paediatric research

## Abstract

Pediatric oncology patients are at high risk of developing clinical deterioration and organ dysfunction during their illness. Heart rate variability (HRV) measured using electrocardiography waveforms is associated with increased organ dysfunction and clinical deterioration in adult and pediatric patients in the intensive care unit (ICU). Here, we explore the feasibility of using photoplethysmography (PPG)-derived integer pulse rate variability (PRVi) to estimate HRV and determine its association with organ dysfunction in pediatric oncology patients in the ward and pediatric ICU. The advantage of using PPG sensor data over electrocardiography is its higher availability in most healthcare settings and in wearable technology. In a cohort of 38 patients, reduced median daily PRVi was significantly associated with increase in two pediatric organ dysfunction scores after adjusting for confounders (*p* < 0.001). PRVi shows promise as a real-time physiologic marker of clinical deterioration using highly-available PPG data, but further research is warranted.

## Introduction

Up to 38% of pediatric oncology patients will be admitted to a pediatric intensive care unit (PICU) within 3 years of their cancer diagnosis due to organ dysfunction, which can result in high morbidity and mortality.^1^ Improving our ability to detect clinical deterioration earlier could improve outcomes.

Reduced heart rate variability (HRV) derived from electrocardiographic waveform data is associated with increased risk of clinical deterioration.^2,3^ Integer heart rate variability (HRVi), derived using intermittent beat-per-minute measurements, has been shown to be an effective surrogate of HRV, even though it requires significantly less data than continuous waveform analysis.^4,5^ However, calculating HRVi requires electrocardiography data, which is harder to acquire and less available than photoplethysmography (PPG) data, particularly in children outside the PICU.^6,7^

The value of using PPG-derived integer pulse rate data to estimate HRV has not been previously reported. Therefore, we aimed to explore the feasibility of using integer pulse rate variability (PRVi) as a surrogate of HRV and to determine its association with two pediatric organ dysfunction scores in oncology patients admitted to the ward and PICU of a children’s hospital.

## Results

**Fig. 1:**
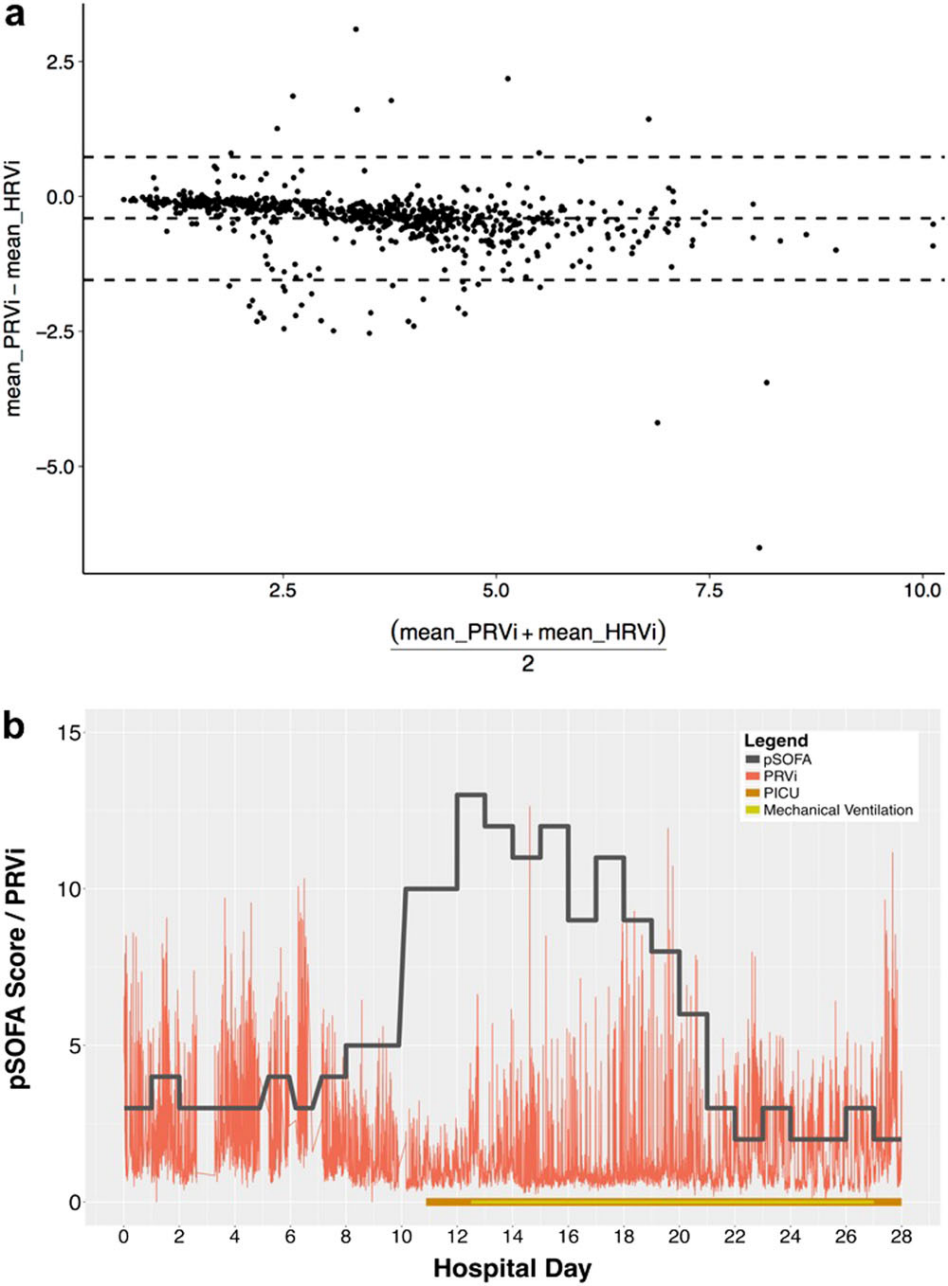
Bland-Altman plot and time series example. Bland-Altman plot (**a**) showing the agreement between mean hourly PRVi and mean hourly HRVi when both measurements were available in this cohort of patients. The limits of agreement, depicted as dashed lines above and below the middle line, were calculated as mean of difference ± 1.96*s, where *s* is the standard deviation of the difference. Time series (**b**) of a patient transferred to the pediatric intensive care unit (PICU) who required mechanical ventilation. The red lines represent the integer pulse rate variability (PRVi). Gaps in the PRVi represent times when the patient was not connected to a PPG sensor. The daily pSOFA score is shown using the dark grey line. Days in the PICU (orange) and on mechanical ventilation (yellow) are shown in the horizontal axis. The patient has a drop in PRVi starting on day 7, which precedes a transfer to the PICU on day 11 and an increase in the pSOFA score. The PRVi reaches its lowest point on days 11 and 12 preceding the initiation of mechanical ventilation

We collected 3.2 million pulse rates during 202 patient-days in 38 patients (median age 8.7 years). A total of 79% of measurements were made outside the PICU. There were 55,075 5-minute intervals with sufficient PPG data, of which 39,650 (72%) also had electrocardiography data. When both measurement were available, mean hourly PRVi had a strong correlation with mean hourly HRVi (*r* = 0.94, *p* < 0.001). Based on the Bland Altman plot, PRVi slightly overestimated HRVi, however, the bias was low at about 1 beat-per-minute above and below the mean (Fig. 1a). Median daily PRVi had a moderate-to-strong negative correlation with the daily pSOFA and PELOD scores across age groups (Pearson’s *r* = −0.6 to −0.7, *p* < 0.001). Reduced median daily PRVi was significantly associated with increased daily pSOFA and PELOD scores after controlling for confounders (both *p* < 0.001; Table 1). For illustration purposes, a time series for a patient who required admission to the PICU and mechanical ventilation is presented (Fig 1b).

**Table 1:** Linear regression analysis of the unadjusted and adjusted association between the median daily PRVi with daily pSOFA and daily PELOD scores

	Daily pSOFA beta coefficients (SE)	*p*	Daily PELOD beta coefficients (SE)	*p*
Unadjusted				
Median daily PRVi, bpm	−1.44 (0.17)	<0.001	−3.43 (0.61)	<0.001
Age-adjusted				
Median daily PRVi, bpm	−1.96 (0.17)	<0.001	−4.31 (0.70)	<0.001
Age, months	−0.01 (0.00)	<0.001	−0.02 (0.01)	0.01
Multivariable				
Median daily PRVi, bpm	−0.96 (0.15)	<0.001	−2.21 (0.63)	<0.001
Median daily pulse rate, bpm	0.01 (0.01)	0.38	0.00 (0.03)	0.98
Lowest SBP, mmHg	−0.02 (0.01)	0.02	−0.30 (0.04)	<0.001
Highest BUN level, mg/dl	0.11 (0.02)	<0.001	0.31 (0.08)	<0.001
Highest serum lactate, mmol/l	2.60 (0.45)	<0.001	7.24 (1.92)	<0.001
Location				
Oncology ward	Reference		Reference	
PICU	0.23 (0.35)	0.51	−2.60 (1.51)	0.09
Age, months	−0.01 (0.00)	<0.001	−0.01 (0.01)	0.43

*pSOFA* pediatric organ failure assessment, *SE* standard error, *PELOD* pediatric logistic organ dysfunction score, *PRVi* integer pulse rate variability, *bpm* beats-per-minute, *SBP* systolic blood pressure, *BUN* blood urea nitrogen, *PICU* pediatric intensive care unit

## Discussion

Reduced PRVi, a new surrogate of HRV, is associated with increased organ dysfunction in pediatric oncology patients in the ward and in the PICU. After adjusting for possible confounders, a drop in the median PRVi by 1 beat-per-minute is associated with an increase in pSOFA score by one point and PELOD score by two points.

Reduced HRV has previously been associated with infection, subsequent clinical deterioration, organ failure, and mortality in children and adults.^2–4,8^ Studies of HRV have largely been limited to the critical care setting, in part due to the limited availability of continuous electrocardiographic data outside the ICU. Furthermore, a recent review of new monitoring technologies for pediatric organ dysfunction highlights the challenges of using electrocardiographic data for estimating HRV.^7^

PRVi is measured using non-invasive PPG, a sensor technology that is ubiquitous in the healthcare setting and increasingly available in wearable technology. This makes PRVi a highly accessible and available digital measure of clinical status, as opposed to other measures of HRV.^6,7^ In addition, the down-sampling involved with the intermittent data needed for calculating PRVi makes it computationally more feasible and generalizable than calculating HRV using continuous waveforms.

Our study was focused on exploring the feasibility of using PRVi in a relatively small cohort of pediatric oncology patients. Further validation in a larger cohort of high-risk patients in other settings, potentially including home monitoring with wearable technology, is warranted.

## Methods

We collected electrocardiography-derived heart rate and PPG-derived pulse rate data every 5 s from monitors (Philips IntelliVue MX800) of 50 beds in the oncology ward and PICU of an academic children’s hospital between 2/2017-4/2017. The 5-second capture interval was chosen based on evidence from prior adult studies.^4,5^

HRVi was calculated as the standard deviation of the heart rate over 5 min using beat-per-minute measurements derived from electrocardiography data, using previously published criteria.^4^ PRVi was calculated in a similar manner but using PPG-based measurements. The 5-minute intervals with < 30 measurements were discarded (<5.3% of available intervals).^5^ Clinical data from the electronic health record was used to calculate the daily pediatric sequential organ failure assessment (pSOFA) score and the daily pediatric logistic organ dysfunction (PELOD) score.^9,10^ All measures were obtained from admission until discharge or hospital day 28, whichever came first.

The mean hourly PRVi was compared to the mean hourly HRVi, when both were available, using Pearson’s coefficient and the Bland Altman plot. The median daily PRVi was compared to the daily pSOFA and the daily PELOD scores using Pearson’s coefficient across three age groups (preschool [2–5 years], school-age [6–11 years], and adolescent [>11 years]), to account for age-dependent heart rate variation. Linear regression was performed to determine the association between PRVi and the two pediatric organ dysfunction scores after controlling for possible confounders. Analyses were performed using standard code in R, version 3.1 (R Project for Statistical Computing).

This study was approved by The University of Chicago Institutional Review Board with waiver of informed consent (IRB#16-1192).

### Data availability

The datasets generated and analyzed for this study are not publicly available due to Institutional Review Board and Health Insurance Portability and Accountability Act restrictions given the identifiable nature of the clinical data used. However, after the necessary approvals and on reasonable request, data will be available from the corresponding author.
